# 

**Published:** 1908-11-15

**Authors:** 


CONTENTS

Event and Comment - - - - - - - - 511

Dental Progress as Indicated in Current Events,
*By George C. Bowles, D.D.S.* 544

Tin and Gold, - - - - - *By C. E. Kells, D.D.S.* 577

Dental Friendship, - - - - - *By Dr. C. P. Elder* 579

Indents - - - - - - - - - - 582

Announcements - - - - - - - - - 593

